# Obstructive Sleep Apnea and Periodontal Disease: A Systematic Review

**DOI:** 10.3390/medicina57060640

**Published:** 2021-06-21

**Authors:** Daniela Lembo, Francesco Caroccia, Chiara Lopes, Francesco Moscagiuri, Bruna Sinjari, Michele D’Attilio

**Affiliations:** 1 Department of Innovative Technologies in Medicine & Dentistry, University “G. d’Annunzio” Chieti-Pescara, 66100 Chieti, Italy; danielalembo3@gmail.com (D.L.); fcaroccia20@gmail.com (F.C.); chiaralopes17@gmail.com (C.L.); francesco.moscagiuri@unich.it (F.M.); b.sinjari@unich.it (B.S.); 2 Department of Surgery and Translational Medicine, University of Florence, 50127 Firenze, Italy; 3 Electron Microscopy Laboratory, University “G. d’Annunzio” Chieti-Pescara, 66100 Chieti, Italy

**Keywords:** obstructive sleep apnea, periodontal disease(s)/periodontitis, periodontal medicine, oral-systemic disease(s), gingivitis, inflammation

## Abstract

*Background and Objectives*: The objective of this study was to evaluate the association between periodontal disease and obstructive sleep apnea syndrome (OSAS). *Materials and Methods*: Electronic search using PubMed, Scopus, LILACS, and Cochrane library was carried out for randomized controlled trials, cohort, case-control, longitudinal and epidemiological studies on humans published from January 2009 until September 2020. The participants had to be male and female adults who were diagnosed with OSAS either by overnight polysomnography (carried out at a sleep laboratory or at home) or by a home sleep testing monitor (Apnea Risk Evaluation System). Methodological quality assessment was carried out using the Newcastle-Ottawa Quality Assessment Scale (NOS) for case-control studies while an adapted form of NOS was used for cross-sectional studies. *Results*: Ten studies fulfilled the inclusion criteria of our review, 5 were case-control studies, and 5 cross-sectional. Sample size ranged from 50 to 29,284 subjects, for a total of 43,122 subjects, 56% of them were male, their age ranged from 18 to 85 years old. The heterogeneity among the studies regarding the classification of periodontal disease, and the different methods for OSAS severity assessment, complicated the comparison among the studies. *Conclusions*: There is low evidence of a possible association between OSAS and periodontitis. The pathophysiological mechanism, cause-effect, or dose-response relationship are still unclear. Further studies are needed and should use a precise classification of OSAS subjects, while the new classification of periodontitis from the World Workshop of Chicago 2017 should be used for the periodontal assessment.

## 1. Introduction

### 1.1. Background

Obstructive sleep apnea syndrome (OSAS) is a chronic sleep-related breathing disorder affecting at least 2–4% of middle-aged females and males [1] while its prevalence is about 2% in children from 2 to 8 years of age [2]. It is characterized by a partial or total obstruction of the upper airways resulting in a reduction of airflow during sleep. This leads to lower oxygen saturation and sleep disruption. Excessive daytime sleepiness, lack of concentration, and snoring are common symptoms of OSAS [1].

OSAS has been associated with an increased risk for the development of cardiovascular disorders such as hypertension, heart failure, coronary heart disease, atherosclerosis, and stroke [3,4]. Furthermore, it has been associated with metabolic disorders, like impaired glucose tolerance and insulin resistance, and depression, cognitive dysfunction, and Alzheimer’s disease [5,6]. Studies have suggested that OSAS could have a role in the activation of various inflammatory processes through hypoxia and oxidative stress-induced reperfusion injury [7,8].

Periodontitis is a chronic disease resulting from the interactions of the host’s defense mechanisms with the pathogenic microorganisms mainly gram-negative bacteria and spirochetes in the biofilm of dental plaque [9]. It is characterized by the destruction of teeth supporting tissue that is clinically evident as pocket formation, the progressive loss of periodontal attachment, the loss of alveolar bone, and eventually tooth loss [10].

Definite risk factors for periodontitis are incorrect oral hygiene [11], genetic pattern [12], diabetes, and smoking [13]. Periodontitis has been associated with various systemic conditions [14] like cardiovascular disease [15], obesity, diabetes mellitus [16], rheumatoid arthritis [17], and pregnant women whose children are born prematurely and with low birth weight [18]. Periodontitis also induces systemic inflammation and oxidative stress [19,20].

Given the common inflammatory nature underlying the two pathologies, it is possible to hypothesize a possible existing association. According to our research, currently, there are only two systematic reviews [21,22] in the literature that investigate this topic, of which the only one with meta-analysis [21]. Al-Jewair et al. [21] in 2015 observed a plausible association between periodontal disease and OSA and wished for further case-control investigation about this topic to draw more valid conclusions. Charles Tremblay et al. [22] in 2016 reported a possible link between periodontitis and OSA syndrome via plasma and salivary inflammatory markers, and once again underlined the need for more studies to clarify the exact relationship between these two conditions.

Since 2016 some studies have been published regarding this topic, therefore arises the need for a new systematic review of the literature.

### 1.2. Objective

The purpose of this study is to evaluate the evidence of a possible association between periodontal disease and OSAS through a systematic review of the literature to answer the following questions:

- Is there an association between OSAS and periodontitis?
- Does OSAS increase the risk of periodontitis?
- Does the severity of periodontitis increase as the severity of OSAS rises?
- Is there a causal relationship between OSAS and periodontitis?

## 2. Materials and Methods

### 2.1. Protocol

In undertaking this systematic review, a search and selection strategy was developed by following standards and guidelines reported in the PRISMA Statement [23].

### 2.2. Eligibility Criteria

The studies were included or excluded in this review using the PICOS (participants, intervention, comparisons, outcomes, study design) criteria.

Randomized controlled trials, cohort, case-control, longitudinal and epidemiological studies were all considered while case reports, case series, reviews, books or book chapters, abstracts, editorials were excluded. Only studies on humans were included, the participants had to be male and female older than 18 years old who were diagnosed with OSAS either by overnight polysomnography, carried out at a sleep laboratory or at home, or by home sleep testing monitor—Apnea Risk Evaluation System (ARES). Whereas studies in which subjects were assessed to be at high risk of OSAS using questionnaires such as Epworth Sleeping Score (ESS), Berlin Questionnaire, STOP or STOP BANG were excluded. Studies with children or with Down syndrome subjects were also excluded. Studies assessing either clinical and radiographic periodontal indices and salivary biochemical analysis were included.

### 2.3. Information Sources and Search Strategy

**Figure 1:**
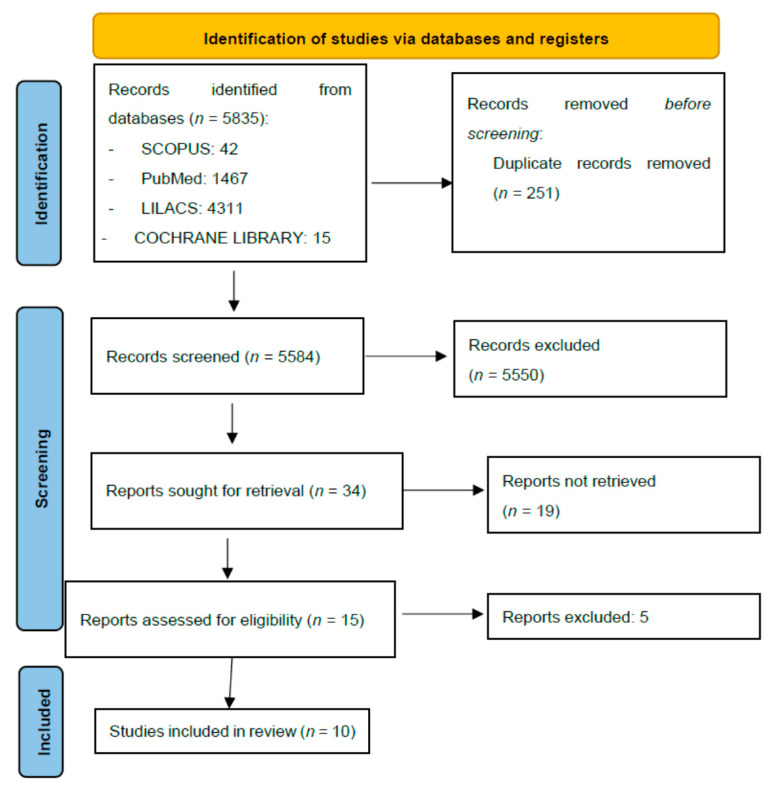
PRISMA flow chart.

The following databases were searched: PubMed, Scopus, LILACS, and Cochrane library (Figure 1). Keywords employed were the same in each database and were all related to periodontitis or OSAS; key terms were the following: “OSAS” or “OSA” or “OSAHS” or “sleep apnea” or “sleep disordered breathing” or “sleep disorders” and “periodontitis” or “periodontal disease” or “gingivitis” or “oral health”. The research covered the period from January 2009 until September 2020. No language restrictions were applied.

### 2.4. Study Selection

Two independent reviewers (D.L. and F.C.) performed various screens on the initial collection of articles. A first screen was performed by examining titles and abstracts. Subsequently, full texts were assessed for eligibility criteria. Disagreements between reviewers were resolved by discussion until a consensus was reached.

### 2.5. Data Items and Data Collection

Retrieved data were collected into Table 1, reporting the following items: author, year of publication, study design, sample size, middle age, OSAS diagnosis method, AHI, periodontal parameters, results, risk of bias, and authors’ conclusions. Data were extracted by two reviewers (D.L. and F.C.) without blinding. Once again, disagreements between reviewers were resolved by discussion.

### 2.6. Risk of Bias in Individual Studies

The risk of bias in the case-control studies was assessed using the Newcastle Ottawa Scale (NOS) [24] while the cross-sectional studies were assessed using an adapted form of NOS [25].

### 2.7. Data Synthesis and Summary Measures

OSAS severity was evaluated by apnea-hypopnea index (AHI). Periodontitis indices such as Clinical Attachment Level (CAL), Pocket Depth (PD), Alveolar Bone Loss (ABL), Recession (REC) or oral hygiene indices such as Bleeding on Probing (BoP), Periodontal Index (PI), Gingival Index (GI), or salivary cytokines were used to assess periodontal disease. Prevalence and Odds Ratio (OR) were used to investigate a possible relation between the two conditions.

## 3. Results

### 3.1. Study Selection

The search strategy resulted in 5835 studies (Figure 1). After duplicates were removed, 5584 results were processed furtherly. After the exclusion of the articles that did not respond to our questions for title or abstract, 15 studies underwent full-text analysis. Of these full-texts, 5 studies were excluded for mismatch with our outcomes and inclusion criteria, so only 10 studies were finally included.

### 3.2. Study Characteristics

10 studies fulfilled the inclusion criteria of our review, 5 were case-control studies [26,27,28,29,30] and 5 cross-sectional [31,32,33,34,35]. Study characteristics are summarized in Table 1.

### 3.3. Risk of Bias in Individual Studies

The risk of bias in the case-control studies was assessed using the Newcastle Ottawa Scale (NOS) [24] and resulted in 3 studies [26,27,29] with a high risk of bias (5 points) and two studies [28,30] with a medium risk of bias (6 points). In cross-sectional studies, the risk of bias was assessed using an adapted form of NOS [25] and resulted in two studies [34,35] with a low risk of bias (8 points) and three studies with a medium risk of bias (2 studies [31,32] 7 points and one [33] study 6 points). The risk of bias was reported in Table 1.

### 3.4. Results of Individual Studies

Sample size ranged from 50 to 29,284 subjects, for a total of 43,122 subjects, 56% of them were male, their age ranged from 18 to 85 years old. 2 of the 10 studies came from the USA [32,34], 4 from Turkey [27,28,29,30], and the rest from Australia [33], Columbia [31], Korea [35], and Taiwan [26].

All the studies evaluated the association between periodontal disease and OSAS and one [26] specifically evaluated the efficacy of periodontal treatment on the severity of OSAS. All studies used an overnight polysomnography (PSG) performed in a sleep laboratory or even at home for OSAS diagnosis, except for one [34] that used the home sleep testing monitor (ARES). The apnea-hypopnea index (AHI) was used in all the studies as objective measures to diagnose and measure the severity of OSAS, except one study [33] where more parameters including Oxygen Desaturation Index (ODI), minimum saturation oxygen levels, total sleep time, and mean oxygen saturation level were used

Selection between OSAS and non-OSAS subjects was made in different ways: three studies [26,29,30] just identified OSAS and non-OSAS subjects while all the other studies [27,28,31,32,33,34,35] classified the subjects according to the severity of OSAS based on AHI, however, this division is not uniform.

Primary periodontal outcomes analyzed differed between studies, though most of the studies used Clinical Attack Level (CAL), Pocket Depth (PD), Recession (REC), and oral hygiene indices like Bleeding on Probing (BoP) and Periodontal Index (PI). A few authors analyzed radiographical alveolar bone loss (ABL) and salivary cytokines to ascertain the presence of periodontitis.

Seven studies [26,28,30,31,33,34,35] evaluated the association between OSAS and periodontal disease, reporting that the prevalence of periodontitis is higher in the OSAS group than in the control group. The resulted prevalence of periodontitis in OSAS subjects among the study ranged from 33.8% [26] to 96.4% [30]; while the OR of periodontitis in the OSAS subjects ranged from 1.37 to 1.84.

CAL was correlated with OSAS severity indicators like AHI, ODI, minimum saturation oxygen level, and total sleep time.

In their study, though, Loke et al. [32] did not demonstrate any significant relation between AHI level and % of BoP or sites with CAL ≥ 3 mm, whereas in the same study an association between AHI and plaque percentage was found. Furthermore, CAL was associated in a statistically significant way with levels of IL-1β and hs-CRP in the crevicular fluid and levels of salivary IL-21.

Latorre et al. [31] in 2018 found a significant association between mild OSAS and periodontal disease (OR = 1.37): this association was more likely in women with arterial hypertension or hypertensive cardiomyopathy. The association between mild OSAS and severe periodontitis was also shown in the study by Sanders et al. [34]; the association was strongest in the group of subjects aged 18–34.

The dose-response relationship between periodontal disease and OSAS severity was investigated in two studies [32,35]: just one of them demonstrated a positive association between OSAS and periodontitis or levels of CAL and PD in a dose-response relationship [35]. Loke et al. [32], instead, did not demonstrate the existence of a dose-response relationship and did not even demonstrate an association between OSAS and periodontitis.

Four studies [26,30,31,32] analyzed the role of cytokines within the saliva, crevicular fluid, or serum in the association between periodontal disease and OSAS.

In 2014 Nizam et al. [29] analyzed the salivary concentration of IL-6, IL-33, IL-1β, IL-21 e PTX-33; they found that in the OSAS group IL-6 was significantly lower, while IL-33 was significantly higher. However, no statistically significant difference was found between OSAS and non-OSAS group in the concentration of IL-1β, IL-21 e PTX-33; IL-21 was significantly related with the clinical attachment loss (CAL) [29].

Nizam et al. (2015) [27] evaluated saliva and serum matrix metalloproteinase (MMP), tissue inhibitor of matrix metalloproteinase (TIMP-1), myeloperoxidase (MPO), neutrophil elastase (NE), neutrophil gelatinase-associated lipocalin (NGAL), and the degree of activation of certain metalloproteinases. In this study, they did not find any pathophysiological mechanism that could link the two diseases via neutrophil enzymes or MMPs.

The same authors published in the following year another study [28], in which they saw that there was a change in the composition of microbe in the subgingival plaque particularly in subjects with severe OSAS. They also found that saliva and serum concentrations of IL-6 and apelin were higher in patients with OSAS and that the salivary levels of IL-6 were significantly related to predictors of OSAS severity such as AHI and ODI. Furthermore, the number of apnea episodes was related to CAL.

Gamisiz-Isik et al. (2017) [30] in their case-controlled study found that IL-1β levels in crevicular fluid and high sensibility-C Reactive Protein (hs-CRP) in serum were significantly higher in the OSAS group. There was also a significant association between the levels of hs-CRP in serum and crevicular fluid and PD and CAL and between IL-1β concentration in crevicular fluid and PI, GI, CAL, PD, BoP, and percentage of PD ≥4 mm. The authors, therefore, concluded that OSAS is associated with higher periodontal indices and higher local inflammatory parameters.

## 4. Discussion

Most of the studies selected in this review support the existence of an association between OSAS and periodontal disease. Although there is evidence of a plausible association between OSAS and periodontitis, it should be considered as low evidence and it is still unclear the pathophysiological mechanism that could link these two conditions nor if there is a cause-effect relationship. Furthermore, it is not clear whether there is a dose-response relationship between OSAS and periodontitis, i.e., whether the increased severity of OSAS increases the severity of periodontitis, nor if OSAS increases the risk of periodontitis.

Five out of ten studies [27,28,30,31,34] included in this review were not included in the previous review by Al-Jewair et al. [21] because published after 2015 indicating an increased interest in the scientific community on this topic. Although the number of observational studies has increased in this review, the risk of bias assessment still shows low-quality evidence in most of the studies, thus results should be cautiously interpreted. The comparison between the studies was complicated by the fact that not all researchers used the same definition of periodontitis and that some studies simply distinguished patients in OSAS and non-OSAS while others stratified OSAS patients by disease severity. Moreover, this stratification within the OSAS group seems heterogeneous among the studies. During the World Workshop of Chicago in 2017 [10] a new periodontitis classification scheme has been adopted which subdivided periodontitis according to a multi-dimensional staging and grading system. This system attempts to ensure an objective and uniform classification of the disease and should be used in the investigations to facilitate de comparison among the studies.

During the same workshop, workgroup 3 [14] focused its attention on the systemic diseases and conditions that can affect the course of periodontitis or have a negative impact on the periodontal attachment apparatus, but OSAS was not considered, maybe due to the lack of evidence.

Even if it was not considered in the workshop, an increased prevalence of periodontitis in OSAS patients is likely since these two diseases have some common risk factors and are both underlying systemic inflammatory status. Gunaratnam et al. [33] were the first authors to notice this relationship between the two diseases, demonstrating the presence of a high prevalence of periodontitis in OSA patients (77–79%), although there is no causal link between the two diseases.

Nizam et al. [28] studied if there is an association between periodontitis and OSAS and underlined the presence of a great variety of risk factors in common between the two diseases. In accordance with this evidence, the authors suggest that for future studies it would be preferable to refer to systemic inflammatory pathways in common between the two pathologies to identify any cause-effect link between the same.

Our review, as already mentioned, includes six more articles [27,28,30,31,34] than the previous two [21,22], which add a few more elements to analyze the association between periodontitis and OSAS.

Nizam et al. [29] have found that there is a lower concentration of the serum levels of proMMP-9 in the severe OSAS group compared to the control group, but there are no differences between the mild-moderate OSAS group and the control group. Salivary NE resulted lower in the OSAS group. The authors concluded that there is no pathophysiological link between the severity of OSAS and the periodontal clinical status mediated by the products of neutrophils and MMPs, despite periodontal clinical parameters were higher in the severe OSAS group but the difference between the study groups was not statistically significant.

Nizam et al. [28] noticed that the total of the equivalent CFU averages increase according to the severity of the OSAS, and above all Gram—microorganisms. Salivary levels of IL-6 are significantly higher in the OSAS group than in the non-OSAS group, as well as salivary levels of apelin are higher in the severe OSAS group than in the control group. Moreover, salivary IL-6 is significantly related to the severity of OSAS. This study underlined that there is a marked change in the presence of particular oral and periodontal microorganisms in the subgingival plaque; these data suggest that OSAS has a connection with the development of periodontal inflammation. Increased salivary IL-6 concentration could both cause and impact periodontal disease in patients with OSAS.

Gamsiz-Isk et al. [30] have found that PI, GI, PD, CAL, BoP, PD ≥ 4 mm are significantly higher in the OSAS group. Periodontitis prevalence is 96.4% in the OSAS group compared to 75% in the non-OSAS group. The prevalence of severe periodontitis in the OSAS group is 48.2%, and the levels of IL-1β in GCF and hs-CRP in serum are significantly higher in the OSAS group. Therefore, the authors concluded that periodontitis prevalence is higher in OSAS groups compared to the control group and OSAS is associated with periodontal indices and local inflammation parameters such as higher IL-1β.

Latorre et al. [31] have found a statistically significant association between mild OSAS and periodontitis (OR = 1.37). This association is more frequent in women with hypertension or hypertensive cardiomyopathy, while periodontitis is associated with severe OSAS in men with hypertension or hypertensive cardiomyopathy.

Sanders et al. [34] noticed that severe periodontitis is positively associated with OSAS, and this association is more pronounced in young adults (18–34 age group), while there is no apparent relationship between OSAS and prevalence of severe periodontitis in subjects aged ≥55 years. There is an independent association between severe periodontitis and OSAS but blood levels of hs-CRP do not explain this relationship.

Therefore, these studies confirm the existence of a relationship between OSAS and periodontitis, but there is a high heterogenicity between the studies that make clear the need to clarify the mechanism of interaction between the two conditions.

## 5. Conclusions

There is low evidence of a possible association between OSAS and periodontitis. The pathophysiological mechanism, cause-effect, or dose-response relationship are still unclear. Further studies are sorely needed and should use a precise classification of OSAS subjects and a standardizing method for periodontitis assessment, possibly according to the staging and grading system showed in the World Workshop of Chicago 2017.

## Figures and Tables

**Table 1:** Data extracted from the included studies. C-C: a case-control study, CS: cross-section study. pro-Matrix MetalloPeptidase 9, MMP-8: Matrix MetalloPeptidase 8, TIMP-1: MetalloProteinase Inhibitor 1 Precursor, NE: Neutrophil elastase, MPO: Myeloperoxidase, proMMP-2 pro-Matrix MetalloPeptidase 2, NGAL: Neutrophil Gelatinase-Associated Lipocalin, MMP-9: Matrix MetalloPeptidase 9, IL-1β: Interleukin-1β, GCF: Gingival Crevicular Fluid, hs-CRP: high sensibility-C Reactive Protein, CFU: colony-forming units, IL-6: Interleukin-6, TNF-α: Tumor Necrosis Factor-α, sRANKL: Soluble receptor activator of nuclear factor (NF)-κB ligand, OPG: Osteoprotegerin, SBD: Sleep-related Breathing Disorder, IL-33: Interleukin-33, IL-21: Interleukin-21, PTX-3: pentraxin-3, ODI: Oxygen Desaturation Index, MMPS: Matrix MetalloPeptidases, SpO_2_: oxygen saturation.

Author	Type of Study	Sample Size	Middle Age	OSAS Diagnosis Method	AHI	Periodontal Parameters	Results	Risk of Bias	Authors’ Conclusions
Keller et al. 2013	C-C	29,284 (18,232 M; 11,052 F)	47.6 (±15.4)	PSG	<5 >5	PD (6 sites/each tooth), ABL	Prevalence of periodontitis: in cases 33.8% vs. 22.6% in controls OR of prior periodontitis for cases was 1.75 (95% CI = 1.68–1.88) times greater than that of controls OR = 1.78 of OSAS in patients with previous periodontitis excluding those who received treatment	5 high	There is an association between OSAS and a previous diagnosis of periodontitis
Nizam et al. 2015	C-C	50 (20 F; 30 M)		PSG	<5 5–30 >30	CAL, PD, BoP, PI (all 6 sites/each tooth)	Serum levels of proMMP-9 significantly lower in the severe OSAS group than in the control. No difference between the control group and the mild-moderate OSAS group. No significant difference between the study groups in serum levels of MMP-8, TIMP-1, NE, MPO, proMMP-2, or MMP-8/TIMP-1 ratio. Significantly lower salivary NE concentration in the OSAS groups compared to the control group. No significant difference between groups in the salivary concentration of MMP-8, TIMP-1, MPO, NGAL, or MMP-8/TIMP-1 ratio. The salivary concentration of proMMP-2 significantly lower in the OSAS groups than in the control group. Degree of activation of salivary MMP-9 significantly lower in the severe OSAS group than in the control group. Negative correlation between ODI and serum levels of proMMP-2 and between AHI and serum concentration of NE and proMMP-2. Negative correlation between AHI and salivary proMMP-9 and -2	5 high	There is no pathophysiological link between the severity of OSAS and the periodontal clinical status mediated by the products of neutrophils and MMPs. Periodontal clinical parameters were higher in the severe OSAS group but the difference between the study groups was not statistically significant.
Nizam et al. 2016	C-C	52 (32 M; 20 F)		PSG	<5 5–30 >30	CAL, PD, salivary cytokines BoP, PI (6 sites/each tooth)	The total of the equivalent CFU averages increases according to the severity of the OSAS. Marked increase in Gram—in plaque samples from patients with severe OSAS and periodontal disease. Salivary levels of IL-6 significantly higher in the OSAS group than in the non-OSAS group. Significantly higher salivary apelin levels in the severe OSAS group than in the control group. Salivary levels of TNF-α, sRANKL, OPG, and OPG/sRANKL similar in all study groups. Highest serum IL-6 and apelin concentration in the OSAS group. Salivary IL-6 is significantly related to the severity of OSAS. CAL-related number of apnea episodes	6 medium	There is a marked change in the presence of particular oral and periodontal microorganisms in the subgingival plaque; these data suggest that OSAS has a connection with the development of periodontal inflammation. Increased salivary IL-6 concentration could both cause and impact periodontal disease in patients with OSAS
Nizam et al. 2014	C-C	52 (32 M; 20 F)	46.60	PSG	<5, 5–30 >30	CAL, PI, PD, and BoP (6 sites/each tooth), salivary cytokines	The concentration of IL-6 significantly lower in the control group than in the group with OSAS IL-33 significantly higher in the OSAS group than in the non-OSAS group No statistically significant difference in IL-1β, IL-21, and PTX-3 concentration between OSAS and non-OSAS Significant correlation between CAL and IL-21 PD and CAL significantly correlated with OSAS severity indicators such as AHI, ODI, SpO_2_	5 high	OSAS does not affect the salivary levels of IL-1β, IL-21, and PTX-33 The levels of IL-6 and IL-33 increase in the patients. OSAS regardless of the severity of the OSAS The increased concentration of these cytokines could play a role in the pathogenesis of periodontal disease in patients with OSAS
Gamsiz-Isik et al. 2017	C-C	163 (122 M; 41 F)	45	PSG	<5 5–15 >15	CAL, PD, PI by Silness and Loe, GI (all 6 sites/each tooth), and BoP	PI, GI, PD, CAL, BoP, PD ≥ 4 mm, and PD ≥ 4 mm% significantly higher in the OSAS group. Periodontitis prevalence 96.4% OSAS group compared to 75% non-OSAS. Prevalence of severe periodontitis in the OSAS group 48.2%. Levels of IL-1β in GCF and hs-CRP in serum significantly higher in the OSAS group. Significant correlation between IL-1β in GCF and PI, GI, CAL, PD, BoP, and PD ≥ 4 mm% CAL and PD significantly associated with hs-CRP levels in serum and GCF in the OSAS group	6 medium	Periodontitis prevalence is higher in OSAS groups compared to the control group. OSAS associated with periodontal indices and local inflammation parameters such as higher IL-1β
Latorre et al. 2018	CS	199 (107 F; 92 M)	49.9	PSG	<5 5–15 15–30 >30	CAL, PD (6 sites/each tooth)	Prevalence of periodontitis 62.3% Statistically significant association between mild OSAS and periodontitis OR = 1.37	7 medium	Statistically significant association between mild OSAS and periodontitis. This association is more frequent in women with hypertension or hypertensive cardiomyopathy. Periodontitis associated with severe OSAS in men with hypertension or hypertensive cardiomyopathy
Loke et al. 2015	CS	100 (91 M; 9 F)	52.6	PSG	<5 5–15 15–30 >30	CAL, PD, REC (all 6 sites/each tooth), PI (4 sites/each tooth) and BoP	Prevalence of periodontitis in the sample population 73%. If AHI is expressed as a continuous variable, there is no correlation between AHI and the severity of periodontal disease. Significant relationship between AHI class and % of plaque. No significant relationship between AHI class and % BoP or sites with CAL ≥ 3	7 medium	A statistically significant association was not found between OSAS and the prevalence of moderate/severe periodontitis; no association was found between the severity of OSAS and the periodontal state
Gunaratnam et al. 2009	CS	66 (54 M; 12 F)	54.9	PSG	>5	CAL, PD, BoP, PI by Silness and Loë, REC, GI (modified by Lobene)	Periodontitis prevalence in the OSAS group 77–79%. Significant association between CAL and total sleep time	6 medium	Higher prevalence of periodontitis in OSAS patients than in non-OSAS
Sanders et al. 2015	CS	12,469 (7473 F; 4996 M)		ARES	0 0–5 5–15 >15	CAL, PD, and REC (6 sites/each tooth)	Greater prevalence of periodontitis as the severity of OSAS increases. The association between periodontitis and OSAS is stronger in the 18–34 age group. No apparent relationship between OSAS and the prevalence of severe periodontitis in subjects aged ≥55 years. OR = 1.4 of severe periodontitis in patients with subclinical SDB; in patients with mild SDB OR = 1.6; with moderate/severe SDB OR = 1.5	8 low	Severe periodontitis is positively associated with OSAS, in particular mild. This association is more pronounced in young adults. There is an independent association between severe periodontitis and OSAS. Blood levels of hs-CRP do not explain this relationship.
Seo et al. 2013	CS	687 (460 M; 227 F)	55.85 (±6.63)	PSG	>5	CAL, PD, BoP, PI by Silness and Loë, REC, GI	Prevalence of periodontitis in the whole sample population 17.5%. 60% of subjects with periodontitis had OSAS. OSAS associated with periodontitis OR = 1.84. OSAS associated with periodontitis in the age group ≥55 years OR = 2.51. Dose-response relationship between periodontitis and OSAS severity	8 low	There is a significant association between OSAS and periodontal disease. OSAS positively associated with periodontitis, PD, and CAL in a dose-response manner.

## Data Availability

The data presented in this study are available on request from the corresponding author.
